# Supplementary material for: Agronomical, biochemical and histological response of resistant and susceptible wheat and barley under BYDV stress
Source: PeerJ. 2018 May 28;6:e4833. doi: 10.7717/peerj.4833 (PMC5978399; doi:10.7717/peerj.4833)
Supplement: Table S1 [file peerj-06-4833-s001.docx]

Table S1 Analysis of variance (F-value) for different biochemical parameters of wheat and barley cultivars as affected by main factors (cultivars and treatment) and their interaction

|  |  | **At 3 weeks after inoculation** | | | | **At 6 weeks after inoculation** | | | |
| --- | --- | --- | --- | --- | --- | --- | --- | --- | --- |
| **Parameter** | **Source of variation** | **S.S.** | **D.F.** | **M.S.** | **F** | **S.S.** | **D.F.** | **M.S.** | **F** |
| Chl a | Cultivar | 2.76 | 3 | 0.92 | 4.98* | 11.28 | 3 | 3.76 | 37.54** |
|  | Treatment | 10.76 | 1 | 10.76 | 58.35** | 6.06 | 1 | 6.06 | 60.52** |
|  | Cultivar × Treatment | 1.85 | 3 | 0.62 | 3.34 | 1.14 | 3 | 1.14 | 11.41** |
|  | Error | 2.95 | 16 | 0.18 |  | 1.60 | 16 | 0.10 |  |
| Chl b | Cultivar | 1.37 | 3 | 0.46 | 3.14 | 3.08 | 3 | 1.03 | 11.92** |
|  | Treatment | 3.77 | 1 | 3.77 | 25.98** | 4.26 | 1 | 4.26 | 49.59** |
|  | Cultivar × Treatment | 0.45 | 3 | 0.15 | 1.04 | 1.84 | 3 | 0.61 | 7.14** |
|  | Error | 2.32 | 16 | 0.15 |  | 1.38 | 16 | 0.09 |  |
| Chl ab | Cultivar | 7.18 | 3 | 2.39 | 4.02* | 25.26 | 3 | 8.42 | 34.39** |
|  | Treatment | 29.24 | 1 | 29.24 | 49.05** | 20.48 | 1 | 20.48 | 83.65** |
|  | Cultivar × Treatment | 3.13 | 3 | 1.04 | 1.75 | 9.20 | 3 | 3.07 | 12.52** |
|  | Error | 9.54 | 16 | 0.60 |  | 3.92 | 16 | 0.24 |  |
| Protein | Cultivar | 0.01 | 3 | 0.00 | 0.97 | 0.05 | 3 | 0.02 | 2.37 |
|  | Treatment | 0.03 | 1 | 0.03 | 8.17* | 0.60 | 1 | 0.60 | 86.46** |
|  | Cultivar × Treatment | 0.01 | 3 | 0.00 | 0.80 | 0.13 | 3 | 0.04 | 6.01** |
|  | Error | 0.07 | 16 | 0.00 |  |  |  |  |  |
| Phenol | Cultivar | 1.23 | 3 | 0.41 | 15.32** | 0.59 | 3 | 0.20 | 14.31** |
|  | Treatment | 0.00 | 1 | 0.00 | 0.06 | 0.63 | 1 | 0.63 | 46.18** |
|  | Cultivar × Treatment | 0.03 | 3 | 0.01 | 0.32 | 0.29 | 3 | 0.10 | 7.02** |
|  | Error | 0.43 | 16 | 0.03 |  | 0.22 | 16 | 0.01 |  |
| Sugar | Cultivar | 75.31 | 3 | 25.10 | 102.72** | 1.07 | 3 | 0.36 | 4.20* |
|  | Treatment | 1.86 | 1 | 1.86 | 7.60* | 5.98 | 1 | 5.98 | 70.59** |
|  | Cultivar × Treatment | 0.18 | 3 | 0.06 | 0.25 | 1.82 | 3 | 0.61 | 7.17** |
|  | Error | 3.91 | 16 | 0.24 |  | 1.36 | 16 | 0.08 |  |

*P<0.05, **P<0.01
